# Repurposing approved drugs as Pendrin (*SLC26A4*) inhibitors in allergic asthma: single-cell nomination, structure-based screening and functional validation

**DOI:** 10.3389/fphar.2026.1926844

**Published:** 2026-09-15

**Authors:** Jianxing Lai, Duoduo Zhao, Xuwen Wang, Binben Wang, Jinliang Nan

**Affiliations:** 1 Key Laboratory of Respiratory Disease of Zhejiang Province, Department of Respiratory and Critical Care Medicine, Second Affiliated Hospital of Zhejiang University School of Medicine, Hangzhou, Zhejiang, China; 2 Bone Marrow Transplantation Center of the First Affiliated Hospital, Zhejiang University School of Medicine, Hangzhou, China; 3 College of Information Science and Electronic Engineering, Zhejiang University, Hangzhou, China; 4 School of Mathematical Sciences, Zhejiang University, Hangzhou, China; 5 Department of Cardiology of the Second Affiliated Hospital, School of Medicine, Zhejiang University, Hangzhou, Zhejiang, China

**Keywords:** asthma, drug repurposing, molecular docking, Pendrin (*SLC26A4*), single-cell RNA-seq

## Abstract

Type-2 airway inflammation remodels the airway epithelium and drives mucus-associated transcriptional programs, making the epithelium an attractive source of drug targets in allergic asthma. Reanalyzing GSE193816, a lower-airway single-cell RNA-seq dataset of allergic asthma (AA) and allergic non-asthma control (AC) participants sampled before and after allergen challenge, a systematic epithelial prioritization ranked *SLC26A4*/Pendrin highly among asthma-enriched candidates, consistent with its previously reported induction in asthmatic airway epithelium. *SLC26A4* was epithelial-biased, was strongest in allergen-challenged asthmatic epithelium, and co-varied with an IL-13/mucus program. Building on this nomination, structure-based virtual screening of approved drugs against the niflumic-acid inhibitor pocket of the cryo-EM Pendrin structure (PDB 8SHC) prioritized six chemically diverse, asthma-unrelated candidates predicted to occupy the pocket. In a halide-flux assay using cells co-expressing Pendrin and a halide-sensitive YFP reporter, these drugs produced concentration-dependent, partial inhibition of Pendrin-mediated transport, with deferasirox and lemborexant the most efficacious and exceeding the niflumic-acid positive control. Because inhibition was partial and occurred at high-micromolar concentrations, these data are a preliminary *in vitro* observation, consistent with but not confirming a repurposing hypothesis for Pendrin in allergic asthma, rather than evidence of therapeutic benefit.

## Graphical Abstract

**Figure:**
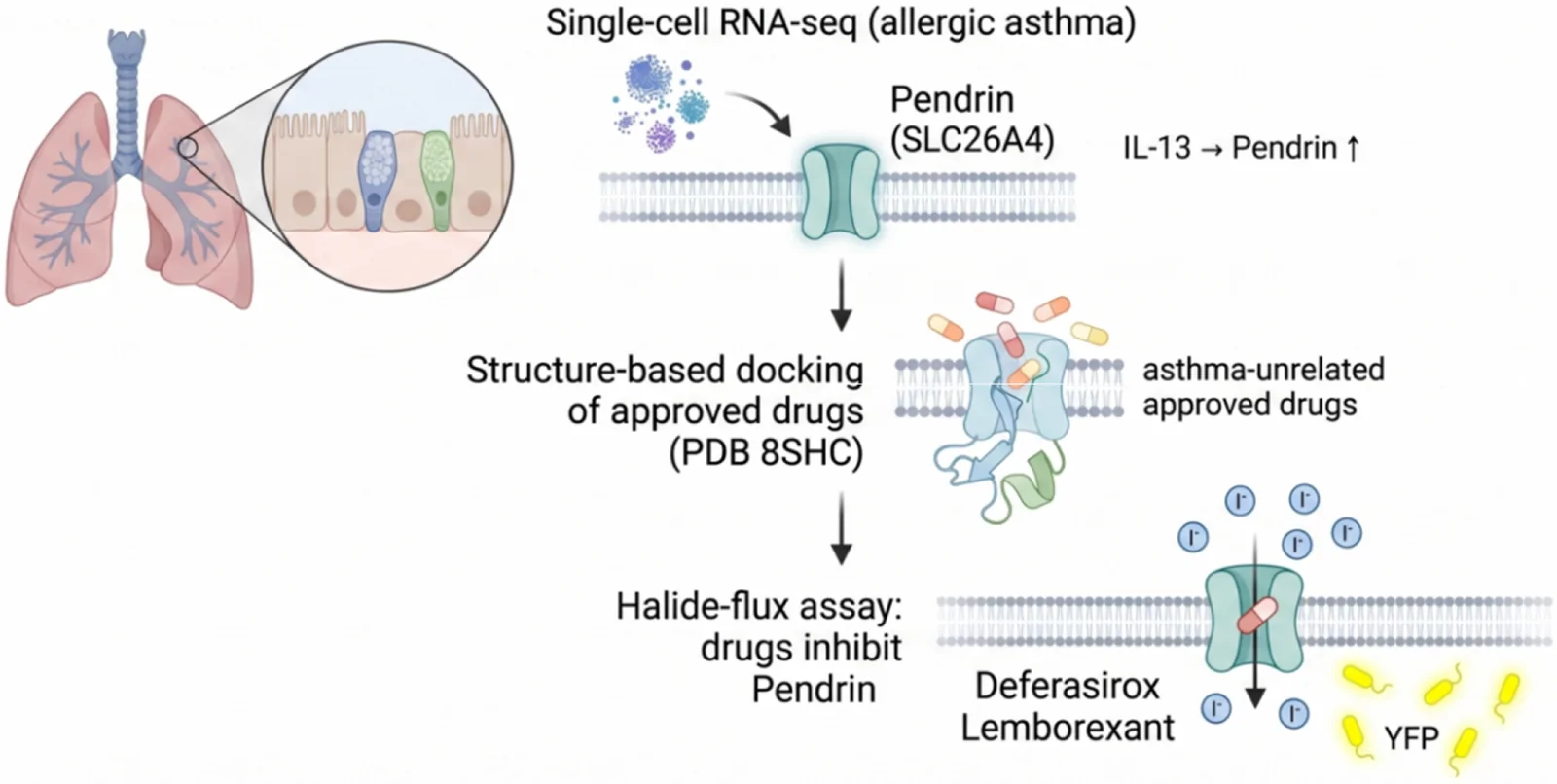

## 1 Introduction

Allergic asthma is a chronic inflammatory airway disease, and in roughly half of patients it follows a type-2-high course driven by the cytokines IL-4, IL-5 and IL-13, with prominent airway hyperresponsiveness and mucus obstruction (Lambrecht et al., 2019; Woodruff et al., 2009). The airway epithelium sits at the centre of this process. Beyond acting as a physical barrier, it senses inhaled allergens, releases alarmins that shape type-2 immunity, and is itself reprogrammed by type-2 cytokines (Lambrecht and Hammad, 2012; Lambrecht and Hammad, 2014; Hammad and Lambrecht, 2015). IL-13 in particular signals through STAT6 and the transcription factor *SPDEF* to push epithelial cells toward goblet-cell metaplasia and excess *MUC5AC* secretion (Kuperman et al., 2002; Park et al., 2007; Chen et al., 2009). Despite the introduction of type-2-targeted biologics, a substantial proportion of patients with asthma remain inadequately controlled, particularly with respect to mucus obstruction, underscoring the need for new therapeutic targets. Because these changes leave a clear transcriptional footprint, the epithelium is an appealing place to look for new drug targets in asthma (Valette et al., 2021).

Pendrin, encoded by *SLC26A4*, is a Cl^−^/anion exchanger that moves bicarbonate, chloride and iodide (Alper and Sharma, 2013; Wang et al., 2024). It is most abundant in the kidney, thyroid and inner ear, where it supports bicarbonate secretion, iodide handling and endolymph ion balance, and loss-of-function mutations cause Pendred syndrome, which combines sensorineural deafness with goitre (Bizhanova and Kopp, 2009; Everett et al., 1997). In the airway epithelium Pendrin is normally low, but IL-4 and IL-13 strongly upregulate it through a STAT6-dependent mechanism (Nakagami et al., 2008; Nofziger et al., 2011). When epithelial Pendrin rises, airway-surface-liquid viscosity increases and mucin release is promoted, and Pendrin is highly expressed in the lungs of asthma-model mice and of patients with asthma and COPD (Nakao et al., 2008; Vanoni et al., 2019; Russo et al., 2017).

These properties have made Pendrin an attractive target. In animal models, genetic or pharmacological reduction of Pendrin attenuates allergic airway inflammation, consistent with its role in driving mucus production in asthma and COPD (Do et al., 2021; Nakao et al., 2008). Yet no Pendrin inhibitor is approved for airway disease. The best-characterised chemical tools, such as niflumic acid (NFA), inhibit Pendrin only at micromolar concentrations. A purpose-built Pendrin inhibitor, YS-01, has been developed and shown to attenuate airway hyperresponsiveness, goblet-cell hyperplasia and mucin expression in an OVA-induced murine asthma model (Park et al., 2019); however, no Pendrin inhibitor is approved for airway disease (Haggie et al., 2016; Cil et al., 2016; Verkman and Galietta, 2009). One way around the cost and time of *de novo* inhibitor development is drug repurposing, in which approved agents are screened for activity against a target so that their established human pharmacology and safety can be leveraged, provided that genuine binders are found and then confirmed functionally (Pushpakom et al., 2019; Yu et al., 2026).

Two recent developments make a structure-guided repurposing approach practical for Pendrin. First, a cryo-EM structure of Pendrin bound to NFA has mapped an inhibitor pocket within the transmembrane anion-permeation pathway and shown that NFA blocks transport by competing with anion binding (PDB 8SHC), giving a defined site for virtual screening (Wang et al., 2024). Second, the public GSE193816 single-cell RNA-seq dataset profiles the lower airway of allergic asthma (AA) and allergic non-asthma control (AC) participants before and after allergen challenge. That design supports a two-axis analysis, comparing allergen response within each disease group and asthma enrichment relative to allergic controls, so that an epithelial target can be nominated from the data rather than assumed in advance.

Prior work has already linked Pendrin to allergic airway disease, including its upregulation in allergen-challenged asthmatic epithelium in the dataset reanalysed here and its IL-13–driven induction in bronchial epithelium (Alladina et al., 2023; Nakao et al., 2008; Nakagami et al., 2008). Our aim is therefore not to identify Pendrin as a new target, but to ask whether a systematic, reproducible prioritization of public single-cell data recovers it among epithelial candidates and, more importantly, whether this nomination can be advanced to structure-based repurposing and functional testing of approved drugs. In this study we combine single-cell transcriptomic prioritization, structure-based drug repurposing and functional validation. We first show that an unbiased epithelial prioritization applied to GSE193816 ranks *SLC26A4*/Pendrin highly among asthma-enriched candidates, consistent with these prior reports. We then dock approved, asthma-unrelated drugs against the Pendrin NFA pocket and test the prioritized candidates in a halide-flux assay. Several of these drugs, led by deferasirox and lemborexant, produce partial, concentration-dependent inhibition of Pendrin-mediated anion transport, providing a preliminary *in vitro* basis, consistent with but not yet confirming, a repurposing hypothesis for Pendrin in allergic asthma.

## 2 Materials and methods

### 2.1 Single-cell reanalysis

#### 2.1.1 Data source and study design

This study used the public GSE193816 single-cell RNA-seq dataset from the human allergic-asthma exacerbation model described previously (Alladina et al., 2023). Two AnnData files were downloaded from GEO: GSE193816_all_cells_data. h5ad (annotated cell-level metadata, UMAP coordinates and visualization) and GSE193816_all_data_raw_counts.h5ad (raw-count normalization and differential expression). The processed object contained 52,152 cells and 28,304 genes, with cell-level metadata for group, condition and cluster (disease group, sampling condition and published cell-type annotation). Disease groups were AA and AC; the primary conditions were baseline (Bln) and allergen challenge (Ag), yielding AC_Bln, AC_Ag, AA_Bln and AA_Ag. Cells from the dilution condition were excluded from the four-group induction and co-expression summaries. Published cluster labels were retained, and no *de novo* clustering, reclustering or relabeling was performed.

### 2.1.2 Normalization and differential expression

Raw-count AnnData was aligned to the processed AnnData by intersecting cell barcodes, and group, condition and cluster metadata were transferred. Raw counts were normalized with scanpy. pp.normalize_total (target_sum = 1e4) and log-transformed with scanpy. pp.log1p. For each annotated cell type and disease group, *SLC26A4* and other genes were compared between Ag and Bln within the same disease group using scanpy. tl.rank_genes_groups (groupby = “condition”, groups = [“Ag”], reference = “Bln”, method = “wilcoxon”); subsets with a missing condition or fewer than 10 cells in the smaller condition were skipped (Wolf et al., 2018). Significant allergen-response DEGs were counted using adjusted *P* < 0.05 and |log_2_ fold change| > 0.5 in the AA Ag-versus-Bln comparison.

### 2.1.3 Epithelial asthma-specific candidate prioritization

Candidate ranking used a differential-expression run with pts = True to retain expression fractions. AA Ag-upregulated genes were filtered by adjusted *P* < 0.05, log_2_ fold change >0.5 and expression in ≥10% of the allergen-challenged target population. To reduce artifacts from near-zero baseline expression, AA and AC logFC values were capped at 5, and the asthma-associated delta was computed as delta_FC = max (logFC_capped_AA− logFC_capped_AC, 0); genes with delta_FC >0.3 were retained. Cell-type specificity was defined as spec_score = max (mean expression across clusters)/sum (mean expression across clusters). A conservative druggability flag was assigned by regular-expression matching of receptor/transporter-like gene families, and the composite target score was delta_FC × spec_score (×1.5 for druggability-flagged genes). *SLC26A4* did not receive the regex-based multiplier, so its ranking derived from delta_FC, epithelial specificity and expression-percentage filtering rather than any hard-coded preference.

### 2.1.4 Specificity, induction and co-expression analyses

*SLC26A4* expression was quantified across all annotated cell types (cell count, mean log-normalized expression, positive-cell fraction and a mean-based specificity score), with UMAP feature plots, violin plots and marker dot plots. For the induction analysis, epithelial cells (cluster = = “Epithelial cells”) were stratified by a concatenated group-condition label (AC_Bln, AC_Ag, AA_Bln, AA_Ag); Mann–Whitney U tests compared log-normalized expression and Fisher exact tests compared positive-cell counts (AA_Ag vs. AA_Bln, AA_Ag vs. AC_Ag, AC_Ag vs. AC_Bln). For co-expression, *SLC26A4* was analyzed with *IL13RA1, CCL26, MUC5AC, SPDEF, CST1* and *POSTN*; single-cell Pearson and Spearman correlations were computed after removing non-finite values.

## 3 Structure-based drug repurposing

### 3.1 Target structure and binding-pocket definition

Docking used the cryo-EM structure of Pendrin from *Sus scrofa* bound to NFA (PDB 8SHC) (Wang et al., 2024). This protein is 780 residues long and aligns gap-free with human Pendrin (UniProt O43511) at 90.9% identity, and the six NFA-pocket residues (Tyr105, Phe141, Phe223, Lys286, Glu303, Asn457) are identical and identically numbered in the human protein. A single protomer (chain C) was extracted, and waters, lipids and detergents were removed. Polar hydrogens were added at pH 7.4 and the receptor was converted to PDBQT with Open Babel (O'Boyle et al., 2011). The docking box was centered on the two co-crystallized NFA molecules of chain C (center 138.98, 118.72, 109.49 Å; size 30.6 × 22.5 × 22.5 Å), defining the validated inhibitor pocket; the chloride-only structure 8SGW was not used.

### 3.2 Compound library and virtual screening

Approved and clinical-stage drugs were obtained from the Broad Drug Repurposing Hub (Corsello et al., 2017). SMILES were desalted (largest organic fragment), neutralized and filtered (molecular weight 120–750 Da; inorganic and metal-containing species removed) with RDKit; 3D conformers were generated with ETKDGv3, MMFF-optimized and converted to PDBQT with Meeko, yielding 4,123 dockable ligands (Riniker and Landrum, 2015; Wang et al., 2020; Halgren, 1996; Eberhardt et al., 2021). Each ligand was docked into the NFA pocket with smina/AutoDock Vina (exhaustiveness 8), with NFA redocked as a positive control (Trott and Olson, 2010; Koes et al., 2013). Candidate triage applied: (i) ranking by docking affinity and ligand efficiency (affinity per heavy atom); (ii) removal of pan-assay-interference, promiscuous and natural-product chemotypes (Baell and Holloway, 2010); (iii) exclusion of classes reported inactive against Pendrin (thiazide diuretics) and the fenamate inhibitor scaffold; and (iv) retention of launched drugs without prior airway or asthma application. From the compounds passing these filters, the top-ranked candidates were inspected individually, and six were advanced to maximize structural and mechanistic diversity, selecting distinct chemical scaffolds and original indications rather than multiple analogues of a single chemotype. Six structurally diverse candidates (deferasirox, dolutegravir, bictegravir, apixaban, ezetimibe, lemborexant) were re-docked at exhaustiveness 16, and the highest-scoring pose of each was retained.

### 3.3 Protein–ligand interaction analysis

Top-pose complexes were analyzed with the Protein–Ligand Interaction Profiler (PLIP) to enumerate hydrophobic contacts, hydrogen bonds, halogen bonds and other interactions, and per-residue interaction fingerprints across the six candidates were tabulated (Salentin et al., 2015). Structural overviews and the labeled lead-candidate pose were rendered with PyMOL; docking-related panels were produced in R (ggplot2, pheatmap).

## 4 Experimental validation

### 4.1 Cell culture and IL-13 stimulation

Immortalized human bronchial epithelial cells (HBE135-E6E7; ATCC, catalogue no. CRL-2741) were cultured in DMEM containing 10% fetal bovine serum (FBS) at 37 °C under 5% CO_2_. For type-2 stimulation, cells were treated with recombinant human IL-13 (50 ng/mL) or vehicle (DMSO) for 48 h.

### 4.2 RNA extraction and RT-qPCR

Total RNA was extracted with TRIzol reagent and reverse-transcribed with PrimeScript reverse transcriptase (Takara). *SLC26A4* mRNA was quantified by RT-qPCR with Bestar SYBR Green qPCR master mix (DBI-2043). The *SLC26A4* primers were forward 5′-TGG​TGG​CTT​GCA​GAT​TGG​AT-3′ and reverse 5′-AGC​TGT​GAG​ACC​AGC​ACT​TG-3′. Expression was normalized to GAPDH and calculated by the 2^(−ΔΔCt)^ method. The GAPDH primers were forward 5′-CGA​CCA​CTT​TGT​CAA​GCT​CA-3′ and reverse 5′-AGG​GGT​CTA​CAT​GGC​AAC​TG-3′.

### 4.3 Flow cytometry for pendrin

IL-13–stimulated and vehicle (DMSO)-treated HBE cells were harvested as single-cell suspensions, fixed and permeabilized (4% paraformaldehyde, 15 min, room temperature, followed by 0.1% Triton X-100, 15 min), and blocked in PBS containing 1% BSA. Cells were stained intracellularly for Pendrin with a rabbit polyclonal anti-Pendrin/*SLC26A4* antibody (Proteintech, 22726-1-AP; 1:100) for 1 h at room temperature, washed, and incubated with a FITC-conjugated goat anti-rabbit IgG (H + L) cross-adsorbed secondary antibody (Invitrogen, F-2765; 1:300) for 30–45 min in the dark. A concentration-matched rabbit IgG isotype control and a secondary-antibody-only control were processed in parallel to define background fluorescence and confirm staining specificity. Flow cytometry was performed on a CytoFlex cytometer (Beckman Coulter, USA). Cells were gated sequentially on FSC-A/SSC-A (cells) and FSC-W/SSC-A (single cells); the Pendrin-positive gate was set against the isotype and secondary-only controls, and events above this threshold were scored as Pendrin-positive. For quantification, the Pendrin mean fluorescence intensity (MFI) of each condition was normalized within each independent experiment to its vehicle (DMSO) control (set to 100%).

### 4.4 Halide-flux functional assay

Although the docking template was the *Sus scrofa* Pendrin structure, the only cryo-EM structure resolving the NFA pocket, the six inhibitor-pocket residues are identical in human *SLC26A4*, and all functional validation was performed on human Pendrin; the computational predictions were therefore tested directly against the human protein. Pendrin transport activity was measured by an iodide-influx assay in HEK293 T cells stably co-expressing human Pendrin (*SLC26A4*) and the halide-sensitive yellow fluorescent protein reporter YFP-H148Q/I152 L (Galietta et al., 2001). Cells were maintained in DMEM supplemented with 10% (v/v) FBS and 1% penicillin/streptomycin at 37 °C under 5% CO_2_, and were seeded at approximately 1 × 10^5^ cells/well in black-walled, clear-bottom 96-well plates 1 2 h before the assay to reach ∼80–90% confluence. On the day of the assay, the culture medium was removed and cells were washed twice with, and then equilibrated for 30–60 min at room temperature in, iodide-free buffer (137 mM NaCl, 2.7 mM KCl, 1 mM CaCl_2_, 1 mM MgCl_2_, 10 mM glucose, 10 mM HEPES, pH 7.4). The six candidate compounds (deferasirox, lemborexant, bictegravir, apixaban, dolutegravir and ezetimibe), the positive-control inhibitor NFA and vehicle (DMSO) were prepared as eight-point serial dilutions in iodide-free buffer spanning 10^–4^ to 10^–11^ M, and were applied to the cells for a 30-min pre-incubation at room temperature. DMSO was carried through the same dilution series as the compounds, and this vehicle series served as the solvent-gradient control. Following a baseline read in iodide-free buffer, an equal volume (100 µL) of iodide-containing buffer was injected into each well to a final extracellular concentration of 70 mM I^−^ (substituted iso-osmotically for Cl^−^), and fluorescence was recorded as Pendrin-mediated iodide entry quenched YFP fluorescence. The per-well readout, Signal Response (%), was defined as [F (compound) − F (DMSO)]/F (baseline) × 100, where F (compound) and F (DMSO) are the post-iodide fluorescence (measured immediately after iodide addition) in the presence of compound or vehicle, respectively, and F (baseline) is the pre-iodide fluorescence. By this definition the vehicle (DMSO) condition is centered at 0%, and higher values indicate greater preservation (rescue) of YFP fluorescence, that is, stronger inhibition of Pendrin-mediated iodide influx. Concentration–response curves were fitted with the log (inhibitor) *versus* response (three-parameter) model in GraphPad Prism 11 to estimate IC_50_.

### 4.5 CCK8 assay

Cytotoxicity was assessed by CCK-8 assay in the HEK293 T cells stably co-expressing Pendrin and the YFP reporter used for the halide-flux assay. Cells in 96-well plates were treated with each candidate compound (deferasirox, lemborexant, bictegravir, apixaban, dolutegravir, ezetimibe), NFA, or vehicle (DMSO) across the same eight-point dilution series used in the halide-flux assay (10^–4^ to 10^–11^ M) for 24 h, with staurosporine (1 µM) as a positive control for loss of viability. CCK-8 reagent was then added and absorbance at 450 nm was recorded. Viability was expressed as a percentage of the vehicle (DMSO) control. n = 3 independent experiments.

### 4.6 Statistical analysis

Data are presented as mean ± SEM as indicated in the figure legends. Statistical analyses were performed in GraphPad Prism 11. Paired comparisons between vehicle (DMSO) and IL-13 conditions from the same cell preparation (Figures 1A,C) were performed using two-tailed paired Student’s t-tests. Single-cell reanalysis statistics (Mann–Whitney U and Fisher exact tests) are cell-level descriptive tests and do not represent donor-level replication. *P* < 0.05 was considered statistically significant (*P < 0.05; **P < 0.01; ***P < 0.001; ****P < 0.0001).

**FIGURE 1:**
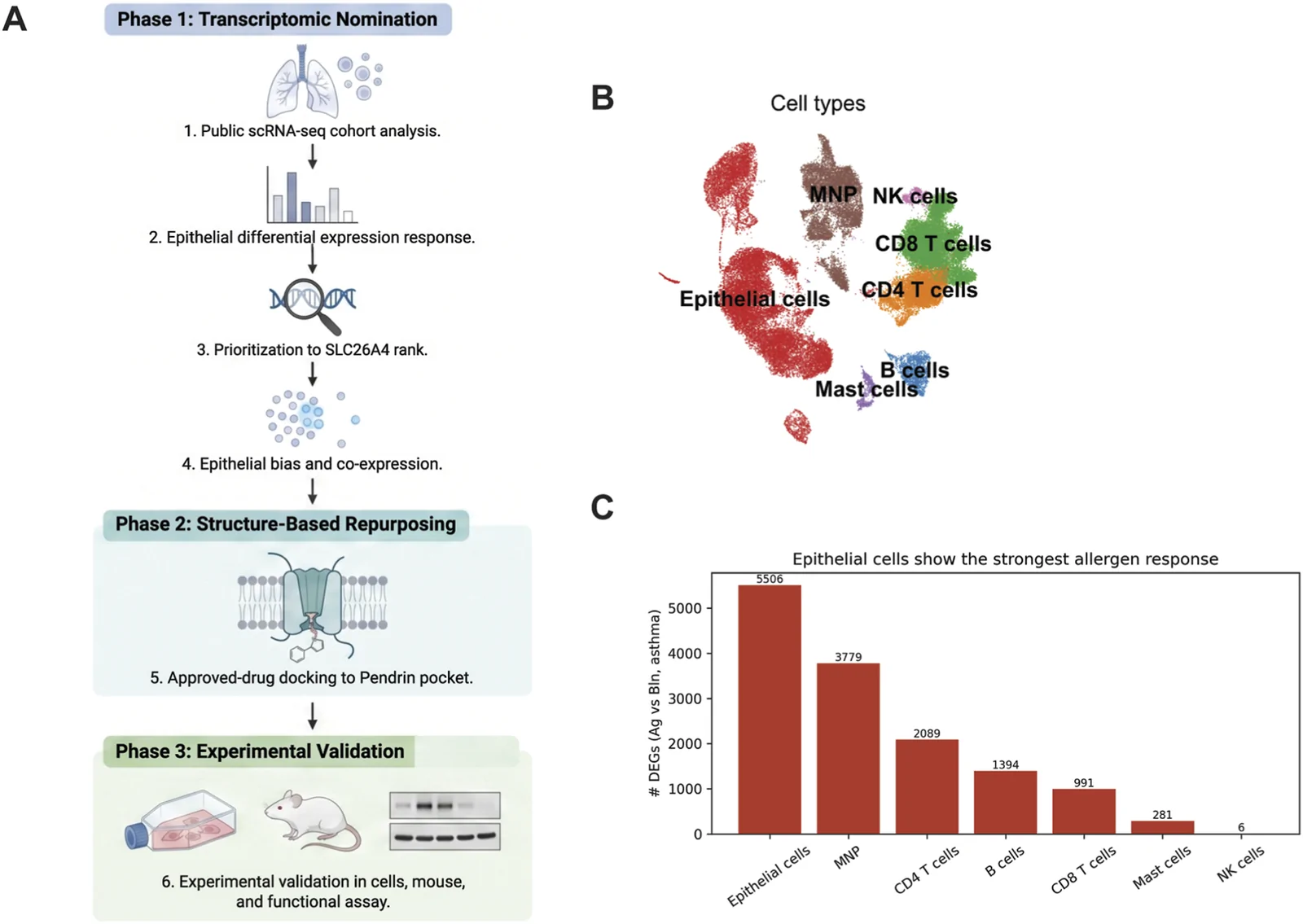
Allergen-challenge response landscape across airway cell types nominates the epithelial compartment. **(A)** Overview of the study workflow: single-cell transcriptomic nomination of *SLC26A4*/Pendrin from the public dataset GSE193816, structure-based repurposing of approved drugs against the Pendrin niflumic acid (NFA) inhibitor pocket, and experimental validation. **(B)** Uniform manifold approximation and projection (UMAP) of the annotated lower-airway single-cell RNA-seq dataset (52,152 cells), coloured by the published cell-type annotation; pre-existing cluster labels and UMAP coordinates were retained. **(C)** Number of significant allergen-response differentially expressed genes (DEGs) per annotated cell type in allergic asthma (AA), computed as allergen challenge (Ag) versus baseline (Bln) (Wilcoxon rank-sum test; adjusted P < 0.05 and |log2 fold change| > 0.5). Epithelial cells show the largest response (5,506 DEGs), followed by mononuclear phagocytes (MNPs; 3,779), CD4 T (2,089), B (1,394), CD8 T (991), mast (281) and NK cells (6).

## 5 Results

### 5.1 Allergen challenge produced the largest transcriptional response in epithelial cells

To identify the airway compartment most responsive to allergen challenge in allergic asthma, we counted significant allergen-response differentially expressed genes (DEGs; adjusted *P* < 0.05, |log_2_ fold change| >0.5) per annotated cell type in the AA Ag-versus-Bln comparison. Cell-type annotations followed the published labels and were supported by canonical lineage markers and quality-control metrics (Supplementary Figure 1) (Zhang et al., 2025). Epithelial cells contained the largest number of allergen-response DEGs (5,506), exceeding mononuclear phagocytes (MNPs, 3,779), CD4 T Cells (2,089), B Cells (1,394), CD8 T Cells (991), mast cells (281) and NK cells (6) (Figure 1; full per-cell-type counts for both AA and AC in Supplementary Figure 1). The epithelial compartment therefore carried the broadest cell-level transcriptional response after allergen challenge, providing a data-driven rationale to prioritize epithelial genes rather than beginning from a predefined immune receptor or cytokine.

### 5.2 Epithelial candidate ranking prioritized *SLC26A4*/Pendrin, which showed epithelial-biased expression

Restricting the analysis to epithelial candidates with an asthma-enriched allergen response, we ranked genes by a composite target score combining the asthma-specific ΔFC (capped AA minus capped AC Ag-versus-Bln logFC), cell-type specificity and expression-percentage filtering. Among 1,338 epithelial candidates, *SLC26A4* ranked seventh by target score and third by asthma-specific ΔFC (logFC 5.288; capped 5.000; AC logFC 1.871; ΔFC 3.129; target score 2.051; adjusted *P* = 6.01 × 10^−15^; AA-Ag expressing fraction 0.137) (Figures 2A,B). Type-2/mucus references including *CCL26, SERPINB3, POSTN* and *CST1* ranked nearby. Notably, *SLC26A4* did not receive the druggability multiplier in the scoring, so its prioritization derived from disease-associated allergen response, epithelial context and expression breadth rather than any hard-coded preference.

**FIGURE 2:**
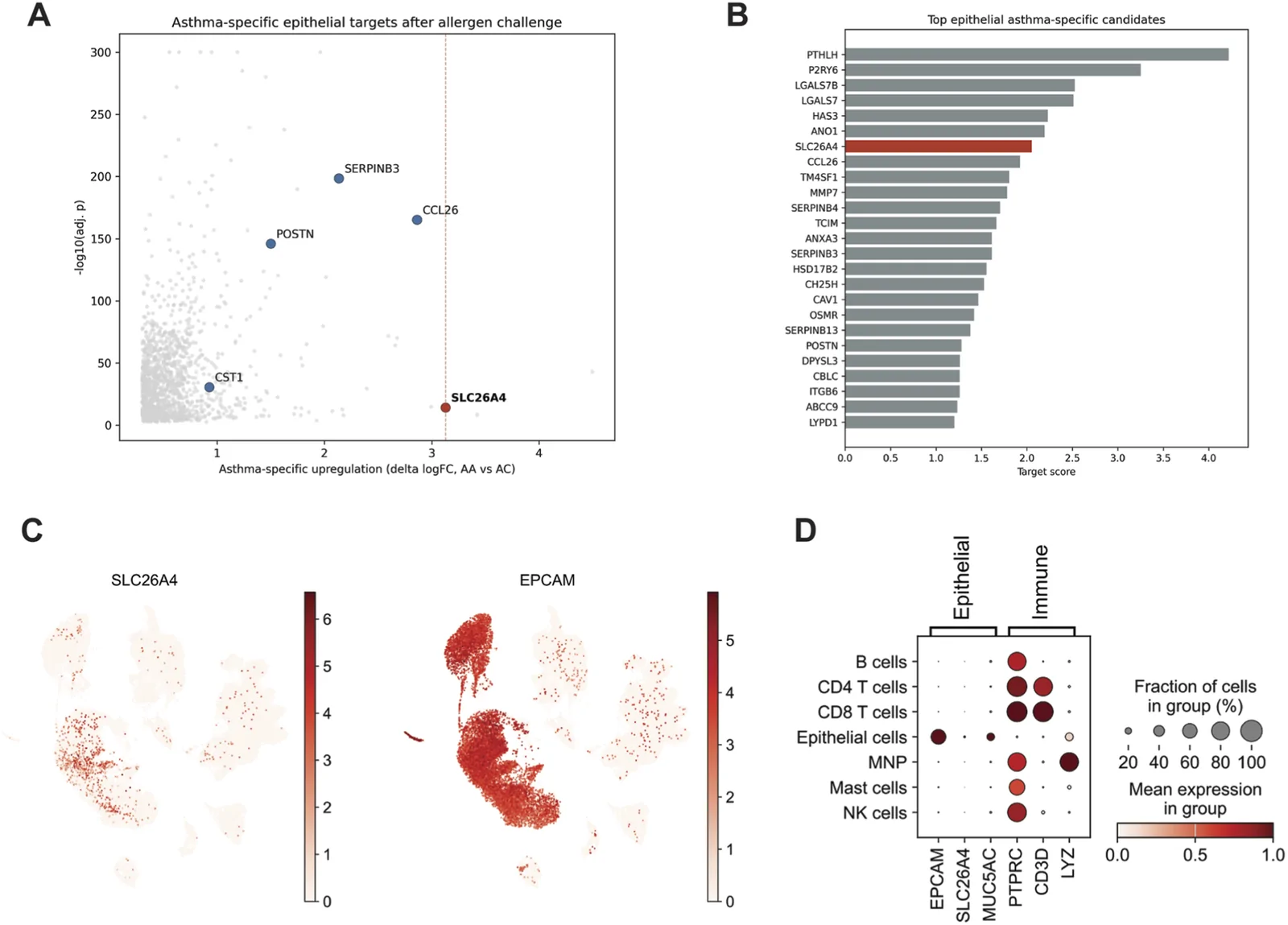
*SLC26A4*/Pendrin is prioritized among epithelial asthma-specific candidates and shows epithelial-biased expression. **(A)** Volcano-style plot of epithelial, asthma-associated allergen-response candidates: asthma-specific ΔFC (capped AA Ag-versus-Bln log2 fold change minus capped AC Ag-versus-Bln log2 fold change) on the x-axis versus −log10 adjusted P on the y-axis; *SLC26A4* and selected type-2/epithelial reference genes are highlighted. **(B)** Top epithelial candidates ranked by the composite target score. Of 1,338 epithelial candidates, *SLC26A4* ranks seventh by target score and third by ΔFC (logFC 5.288; capped logFC 5.000; AC logFC 1.871; ΔFC 3.129; target score 2.051; adjusted P = 6.01 × 10^−15^; AA-Ag expressing fraction 0.137). **(C)** UMAP feature plots of *SLC26A4* and the epithelial marker EPCAM. **(D)** Dot plot of *SLC26A4* across annotated cell types. Epithelial cells (n = 21,069) show the highest mean log-normalized expression (0.108), expressing fraction (0.0373) and mean-based specificity score (0.654), whereas all immune populations show expressing fractions ≤0.0046.

Because a useful epithelial target should not merely appear in an epithelial DEG table, we examined *SLC26A4* across all annotated cell types. Epithelial cells (n = 21,069) showed the highest mean log-normalized *SLC26A4* expression (0.108), positive-cell fraction (0.0373) and specificity score (0.654), whereas all immune populations showed positive-cell fractions ≤0.0046 (Figures 2C,D; Supplementary Figure 2). *SLC26A4* was thus epithelial-biased: when detected, it was concentrated in epithelial cells rather than broadly distributed across immune compartments.

### 5.3 Pendrin was strongest in allergen-challenged asthmatic epithelium and aligned with an IL-13/mucus program

We next asked whether epithelial *SLC26A4* was related to allergen challenge, asthma status, or both, using a four-level group–condition variable. *SLC26A4*-positive epithelial fractions were 0.00829 (AC_Bln), 0.02785 (AC_Ag), 0.00636 (AA_Bln) and 0.14370 (AA_Ag), with mean log-normalized expression following the same ordering (Figures 3C,E). AA_Ag differed from AA_Bln and from AC_Ag; AC_Ag also exceeded AC_Bln but by a smaller margin. *SLC26A4* was therefore allergen-associated, and this increase was markedly stronger in allergic asthma epithelium than in allergic non-asthma controls. These are descriptive cell-level tests and are not donor-level replicates.

**FIGURE 3:**
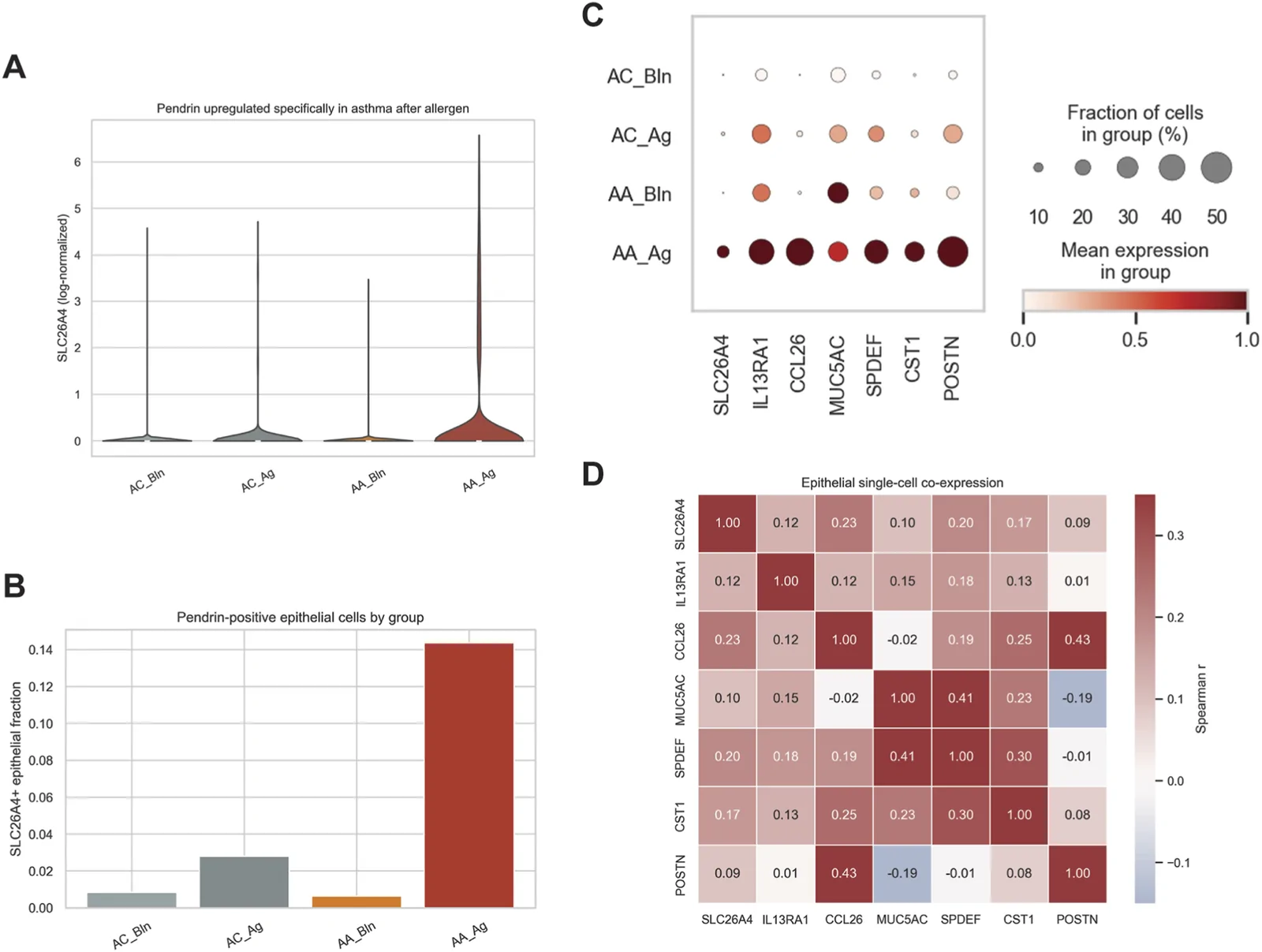
Pendrin is induced in allergen-challenged asthmatic epithelium and co-varies with an IL-13/mucus program. **(A)** Violin plots of epithelial *SLC26A4* expression across the four group -condition strata (AC_Bln, AC_Ag, AA_Bln, AA_Ag). **(B)** Fraction of *SLC26A4*-positive epithelial cells per stratum (AC_Bln 0.00829; AC_Ag 0.02785; AA_Bln 0.00636; AA_Ag 0.14370). Cell-level comparisons: AA_Ag *versus* AA_Bln, Fisher’s exact P = 1.11 × 10^−135^; AA_Ag *versus* AC_Ag, Mann–Whitney P = 2.19 × 10^−34^ and Fisher’s exact P = 5.61 × 10^−39^. These are descriptive single-cell–level tests and are not donor-level replicates. **(C)** Dot plot of *SLC26A4* together with *IL13RA1, CCL26, MUC5AC, SPDEF, CST1* and *POSTN* across the four strata (colour scaled per gene); AA_ Ag shows the highest expressing fraction for *SLC26A4* and most type-2/mucus genes. **(D)** Spearman co-expression heatmap across epithelial cells for the selected gene set; *SLC26A4* correlates positively with *CCL26* (r = 0.230), *SPDEF* (0.197), *CST1* (0.171), *IL13RA1* (0.125), *MUC5AC* (0.103) and *POSTN* (0.094).

To place *SLC26A4* in a type-2/mucus context, we analyzed it alongside *IL13RA1, CCL26, MUC5AC, SPDEF, CST1* and *POSTN*. AA_Ag showed the highest expressing fractions for *SLC26A4* and most of these genes (Figure 3C). Single-cell co-expression was consistently positive, strongest for *CCL26* (Spearman r = 0.230), *SPDEF* (0.197) and *CST1* (0.171), followed by *IL13RA1* (0.125), *MUC5AC* (0.103) and *POSTN* (0.094) (Figure 3D). *SLC26A4* thus aligns with an IL-13/mucus-associated epithelial program without overstating pathway directionality.

### 5.4 Structure-based repurposing nominated approved-drug candidates for the pendrin inhibitor pocket

Having nominated Pendrin/*SLC26A4* transcriptomically, we translated the prioritization into a structure-based repurposing hypothesis using the cryo-EM structure of Pendrin (*Sus scrofa*; PDB 8SHC) bound to NFA. Two NFA molecules define an inhibitor pocket within the transmembrane anion-permeation pathway, lined by residues including Tyr105, Phe141, Phe223, Lys286, Glu303 and Asn457 (Figure 4A). Virtual screening of 4,123 approved/clinical-stage drugs against this pocket, with NFA redocked as a positive control (−8.6 kcal/mol), was triaged by docking affinity and ligand efficiency, removal of promiscuous chemotypes, exclusion of classes reported inactive against Pendrin (thiazide diuretics), and retention of approved drugs without prior airway/asthma application (Figures 4B,C). Six chemically diverse candidates were advanced: deferasirox, lemborexant, bictegravir, apixaban, dolutegravir and ezetimibe, with re-docking affinities of −9.5 to −11.1 kcal/mol, all exceeding the NFA control and led by deferasirox (−11.1 kcal/mol) (Figure 4D). Selection was driven by predicted pocket engagement, independent of the compounds’ original indications.

**FIGURE 4:**
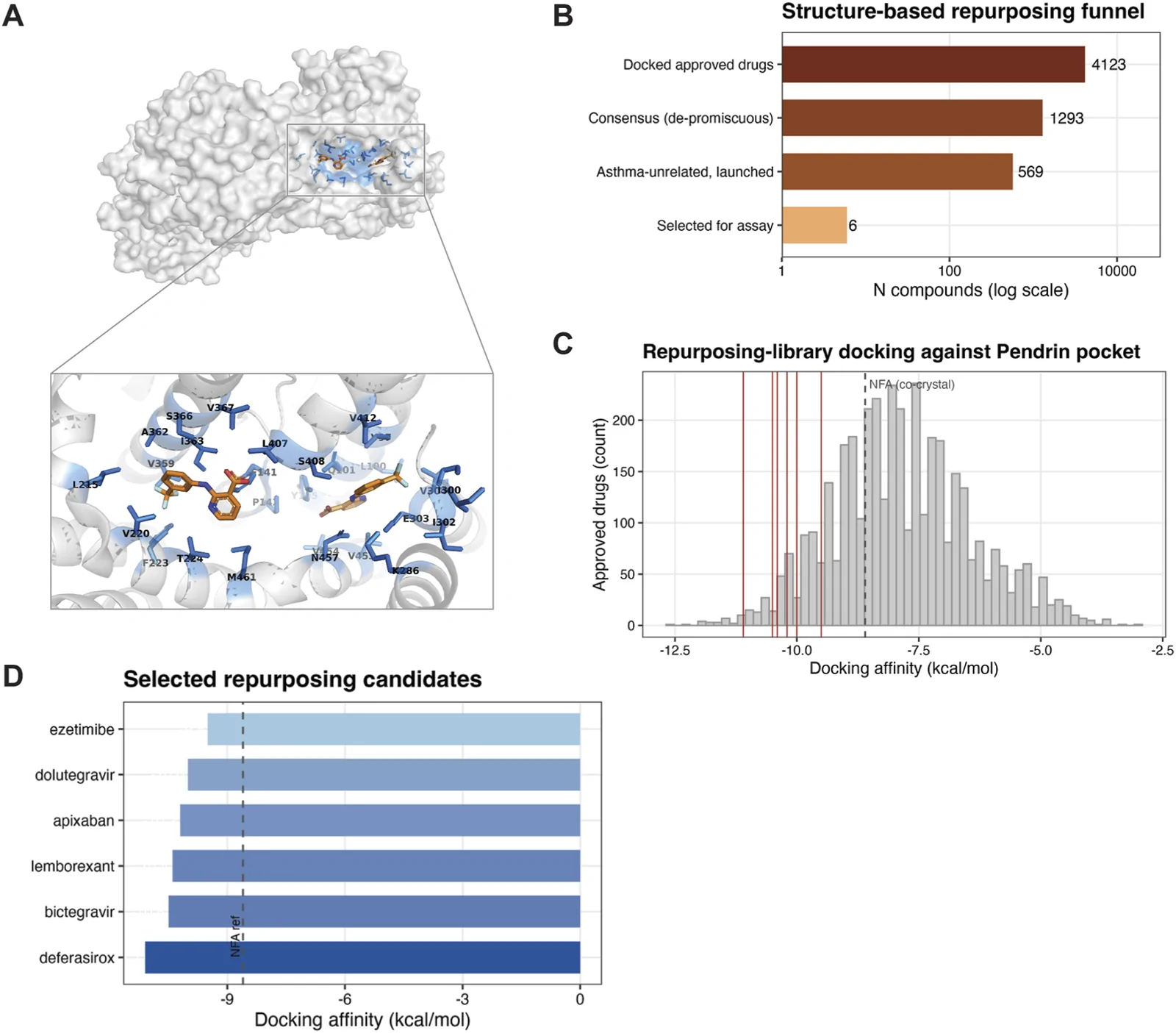
Structure-based repurposing screen against the Pendrin NFA inhibitor pocket. **(A)** Upper: cryo-EM structure of Pendrin (*Sus scrofa*; PDB 8SHC, chain C; grey cartoon) with bound NFA (orange sticks), bound chloride (green sphere) and the pocket-lining residues *Tyr105, Phe141, Phe223, Lys286, Glu303* and *Asn457*. Bottom: close-up of the NFA inhibitor pocket used as the docking target; redocking of NFA recovered the pocket at −8.6 kcal/mol, providing an internal positive control. **(B)** Structure-based repurposing funnel: 4,123 dockable approved/clinical-stage drugs were narrowed through consistency and de-confounding filters and an approved, asthma-unrelated criterion to six candidates. **(C)** Docking-affinity distribution of the approved-drug library, with the redocked NFA control (−8.6 kcal/mol) and the six selected candidates indicated. **(D)** Docking affinities of the six selected candidates, all exceeding the NFA reference, with deferasirox highest (−11.1 kcal/mol).

### 5.5 Predicted binding modes and protein–ligand interaction fingerprints of the candidates

Protein–ligand interaction profiling (PLIP) the docked complexes showed that all six candidates occupied the NFA pocket and recapitulated its characteristic contacts (Figure 5; Supplementary Figure 2). The lead candidate deferasirox formed an extensive network (nine hydrophobic contacts and five hydrogen bonds) with pocket residues including Glu303, Asn457, Tyr105, Lys286 and Ser408 (Figures 5A,B). Binding across the panel was dominated by hydrophobic contacts complemented by hydrogen bonds, with the integrase inhibitors additionally engaging halogen bonds (Figure 5D). The interaction fingerprints converged on a shared set of pocket residues, notably Glu303, Asn457, Gln101, Val97 and Tyr105, indicating that structurally unrelated approved drugs are predicted to engage the same inhibitor sub-pocket (Figures 5C,E). Because docking predicts binding geometry rather than functional inhibition, we next tested these predictions experimentally.

**FIGURE 5:**
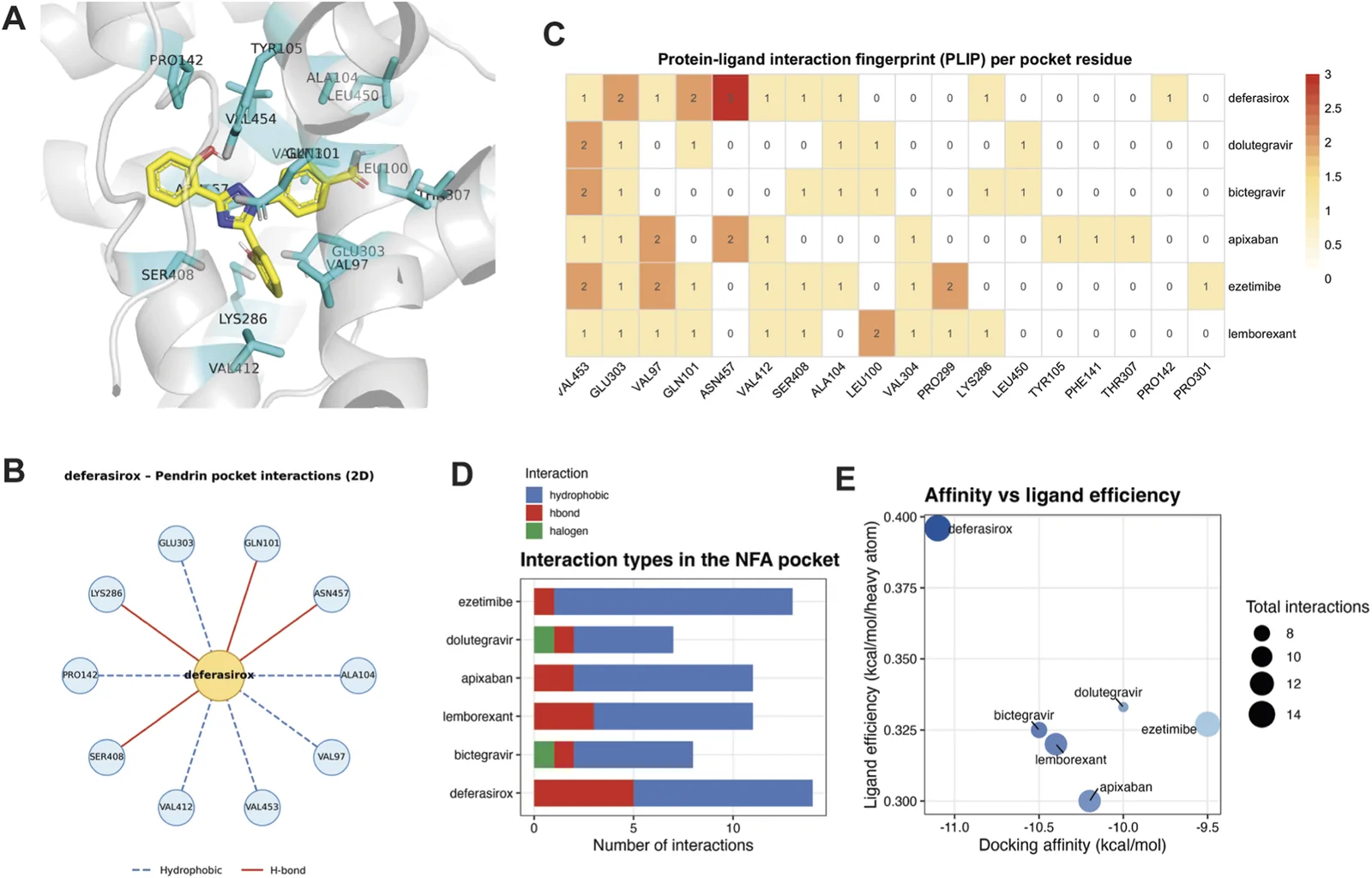
Predicted binding modes and protein–ligand interaction fingerprints of the repurposing candidates. **(A)** Predicted binding pose of the lead candidate deferasirox (yellow sticks) in the NFA pocket, with interacting residues shown as cyan sticks. **(B)** Protein–ligand interaction profiler (PLIP) view for deferasirox (hydrophobic contacts, dashed grey; hydrogen bonds, blue); deferasirox forms nine hydrophobic contacts and five hydrogen bonds with *Glu303, Asn457, Tyr105, Lys286* and *Ser408*. **(C)** Per-residue interaction fingerprints (PLIP) for the six candidates; values are interaction counts per Pendrin pocket residue. Structurally unrelated candidates converge on a shared set of NFA-pocket residues, notably *Glu303, Asn457, Gln101, Val97* and *Tyr105*. **(D)** Interaction-type composition (hydrophobic, hydrogen bond, halogen bond) per candidate. **(E)** Docking affinity versus ligand efficiency; point size encodes the total number of predicted interactions. All interactions are docking-derived predictions requiring experimental validation.

### 5.6 Pendrin is induced in IL-13–stimulated epithelium and is functionally inhibited by repurposed drugs

We first confirmed that Pendrin is induced under type-2 conditions in airway epithelium. In the immortalized human bronchial epithelial cell line HBE135-E6E7, IL-13 stimulation increased *SLC26A4* mRNA relative to vehicle (DMSO) by RT-qPCR (Figure 6A). At the protein level, intracellular flow cytometry for Pendrin showed a marked increase in the Pendrin-positive fraction after IL-13 stimulation (Figures 6B,C). This IL-13–driven induction of Pendrin is consistent with earlier demonstrations in other bronchial epithelial systems and confirms, rather than newly reports, this relationship in the HBE model used here (Nakao et al., 2008; Nakagami et al., 2008). The concordant increase in both the proportion of Pendrin-expressing cells and per-cell Pendrin abundance mirrors the transcriptomic pattern observed in the single-cell data, in which the asthma-enriched *SLC26A4* signal was driven largely by an expansion of the Pendrin-positive epithelial fraction.

**FIGURE 6:**
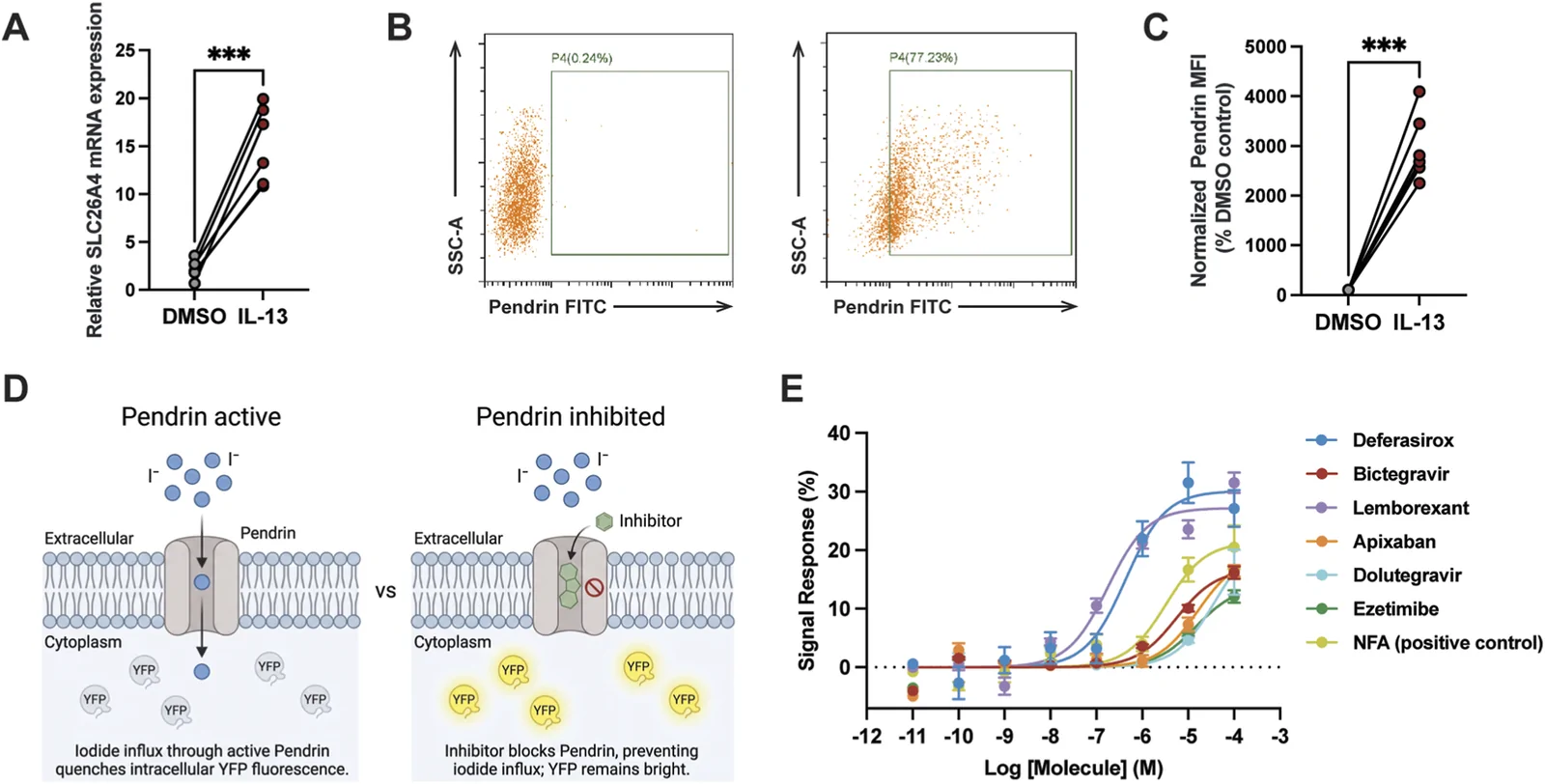
Pendrin is induced in IL-13–stimulated airway epithelium and is functionally inhibited by repurposed approved drugs. **(A)** *SLC26A4* mRNA (RT-qPCR) in human bronchial epithelial (HBE) cells after IL-13 stimulation versus vehicle (DMSO); data are mean ± SEM, n = 6 independent experiments. **(B)** Representative flow-cytometry dot plots of permeabilized HBE cells stained for Pendrin, in vehicle (DMSO; left) and IL-13–stimulated (right) conditions. **(C)** Quantification of Pendrin mean fluorescence intensity (MFI) across independent experiments, normalized within each experiment to its vehicle (DMSO) control (set to 100%); data are mean ± SEM, n = 6 independent experiments. **(D)** Schematic of the halide-flux (iodide) assay: iodide entry through active Pendrin quenches a co-expressed halide-sensitive YFP reporter, whereas an inhibitor blocks Pendrin, preventing iodide influx so that YFP remains bright. Assays were performed in HEK293/293 T cells stably expressing Pendrin together with the YFP reporter. **(E)** Concentration–response curves for the six repurposing candidates (deferasirox, lemborexant, bictegravir, apixaban, dolutegravir, ezetimibe), with NFA as positive control.

We then tested whether the docking-predicted candidates functionally inhibit Pendrin, using an iodide-influx assay in HEK293/293 T cells stably co-expressing Pendrin (Supplementary Figure 3) and a halide-sensitive YFP reporter, in which iodide entry through active Pendrin quenches YFP fluorescence and inhibition preserves it (Figure 6D). All six candidates produced concentration-dependent, partial inhibition of Pendrin-mediated iodide transport. Deferasirox and lemborexant were the most efficacious, exceeding the NFA control, whereas bictegravir, apixaban, dolutegravir and ezetimibe showed weaker responses (Figure 6E). Across the same concentration range, none of the candidates reduced HEK293 T viability at the concentrations producing inhibition, whereas the staurosporine positive control markedly reduced viability, indicating that the observed inhibition is not attributable to nonspecific cytotoxicity (Supplementary Figure 4).

In summary, IL-13 raised Pendrin in airway epithelium at both the transcript and protein levels, and several of the asthma-unrelated drugs nominated by docking, with deferasirox and lemborexant the strongest, inhibited Pendrin-mediated anion transport. This inhibition was only partial and needed high-micromolar concentrations, and the predicted binders differed in activity, which fits the expectation that docking flags binding rather than potency. We therefore regard Pendrin as a workable epithelial target in allergic asthma, with deferasirox and lemborexant as the most promising candidates to pursue.

## 6 Discussion

This study nominated Pendrin/*SLC26A4* as an epithelial drug-development candidate in allergic asthma and then put the resulting repurposing hypothesis to an experimental test. In the transcriptomic data, the epithelial compartment carried the largest allergen response, *SLC26A4* ranked among the top epithelial candidates after expression-percentage filtering and logFC capping, its expression was epithelial-biased and peaked in allergen-challenged asthmatic epithelium, and it tracked with an IL-13/mucus program. Structure-based screening against the validated niflumic-acid (NFA) pocket of Pendrin then prioritized six approved, asthma-unrelated drugs, and a halide-flux assay showed that several of them, with deferasirox and lemborexant the strongest, inhibit Pendrin-mediated iodide transport. The study thus goes past target nomination to a first experimental test of the hypothesis.

Pharmacological inhibition of Pendrin is already a structurally and functionally supported way to attenuate airway pathology, so what is new here is not the target concept but the finding that approved drugs developed for unrelated indications engage and inhibit its pocket (Wang et al., 2024; Do et al., 2021; Nakao et al., 2008; Haggie et al., 2016). The methodological contribution is therefore not the target itself but a reproducible prioritization framework that recovers a known target from public single-cell data without prior assumption. Rather than inspecting individual genes, the framework combines a disease-specificity term (the asthma-enriched allergen response, computed as the difference between capped fold changes in asthma *versus* allergic-control epithelium), a cell-type-specificity term, and expression-breadth filtering into a single composite score, and it does so without any hard-coded preference for a given gene family. Because it depends only on group–condition metadata and standard single-cell outputs, the same scoring approach could be applied to other public single-cell datasets and to other epithelial-driven diseases to nominate compartment-specific, disease-enriched targets. Recovering *SLC26A4*, a target independently supported by prior functional and structural work, provides a positive control for the framework rather than a new biological claim. These compounds carry established safety profiles, which could shorten the path relative to *de novo* inhibitor development if their activity holds up and can be improved. Deferasirox and lemborexant were the strongest of the set.

Our approach is complementary to purpose-built inhibitor development. YS-01, a dedicated Pendrin inhibitor, already attenuates airway hyperresponsiveness and mucin expression in a murine asthma model, establishing Pendrin inhibition as a viable *in vivo* strategy (Park et al., 2019). Rather than designing a new chemical entity, we asked whether existing approved drugs, with established human pharmacology and safety, can engage the same target. Such repurposing candidates, if optimized, could offer a faster translational route, while YS-01 illustrates the potency and *in vivo* efficacy that a developed Pendrin inhibitor can reach and that our high-micromolar hits do not yet approach.

A conceptual question is whether the epithelium, whose remodelling is partly downstream of type-2 immune activation, is an appropriate therapeutic compartment, or whether targeting it risks addressing a consequence rather than a cause. Several considerations bear on this. First, the airway epithelium is not a purely passive downstream tissue: it senses inhaled allergens and releases alarmins such as TSLP, IL-33 and IL-25 that initiate and amplify the type-2 response, so it occupies a bidirectional position in the disease network rather than a strictly downstream one. Second, Pendrin operates at a defined effector step, contributing to mucus hypersecretion and to imbalance of the airway-surface liquid; inhibiting it therefore engages a specific pathological mechanism rather than a nonspecific symptom. Third, and most importantly, we do not position epithelial Pendrin inhibition as superior to, or a replacement for, upstream immune-directed therapies such as anti-IL-4Rα or anti-IL-13 biologics. Rather, it is best regarded as a complementary strategy directed at the mucus-obstruction component of the disease, which remains incompletely controlled in many patients despite current treatments. Whether Pendrin inhibition adds benefit on top of upstream immune blockade, and in which patients, is an open question that will require the functional and *in vivo* studies outlined below.

Our conclusions come with clear limitations. The inhibition we observed was partial, reaching a maximal Signal Response of roughly 30%, so the candidates do not fully block Pendrin-mediated transport under these conditions. This could reflect their modest predicted affinities, incomplete occupancy at the concentrations tested, or a real ceiling of partial inhibition, and telling these apart will need further work. Half-maximal effects fell in the high-micromolar range, which leaves open whether these drugs, at approved doses, reach airway or plasma concentrations able to inhibit Pendrin in a patient. The data are best read as an *in vitro* proof of concept; they do not establish therapeutic exposure or benefit, and reaching pharmacologically relevant potency may require medicinal-chemistry optimization or local airway delivery.

Our functional results should also be read in the context of structure-based virtual screening (SBVS) as a discovery approach. In a survey of 419 prospective SBVS campaigns, only about one-quarter of experimentally confirmed hits reached sub-micromolar potency, and the authors concluded that the principal strength of SBVS is the discovery of new chemotypes rather than of highly potent compounds (Zhu et al., 2022). The partial, high-micromolar inhibition we observe is therefore consistent with the typical output of an initial docking screen, and the candidates are best regarded as starting points rather than optimized inhibitors. Several validations are not yet in place: docking predicts binding geometry rather than potency, and the predicted binders differed in activity, so each compound still requires its own experimental confirmation; the binding poses have not been confirmed by co-crystallography or biophysical assays; and functional inhibition has been demonstrated only in a Pendrin-overexpressing HEK293 T system, not on endogenous Pendrin in differentiated airway epithelium. To exclude nonspecific effects, we confirmed that the candidates were not cytotoxic across the full concentration range tested in the functional assay, whereas a staurosporine positive control markedly reduced viability.

A further caveat is that the single-cell statistics behind the nomination are cell-level descriptive tests rather than donor-level replicates, and the present scope did not include *SLC26A4* perturbation, an *in vivo* model, or an airway-surface-liquid/mucus readout. These gaps point to concrete next steps: confirming inhibition of endogenous Pendrin transport in IL-13–stimulated primary bronchial epithelium, asking whether the lead compounds dampen the downstream type-2/mucus program such as *MUC5AC* and *CCL26*, and, given the high-micromolar potency, exploring structure-guided optimization or localized delivery before any *in vivo* or disease-level test.

## 7 Conclusion

By combining single-cell transcriptomic prioritization, structure-based repurposing and functional validation, this study nominates Pendrin/*SLC26A4* as an epithelial target in allergic asthma and identifies approved, asthma-unrelated drugs that inhibit it, with deferasirox and lemborexant the strongest. IL-13 raised Pendrin in airway epithelium at both the transcript and protein levels, and the lead compounds produced concentration-dependent inhibition of Pendrin-mediated anion transport. The inhibition was partial and occurred at high-micromolar concentrations, so these findings represent an early, partially validated repurposing hypothesis rather than evidence of therapeutic efficacy. They provide a concrete and testable basis for further mechanistic and translational study, beginning with confirmation in primary airway epithelium.

## Data Availability

The datasets presented in this study can be found in online repositories. The names of the repository/repositories and accession number(s) can be found in the article/Supplementary Material.
